# The impact of parental migration patterns, timing, and duration on the health of rural Chinese children

**DOI:** 10.3389/fpubh.2024.1439568

**Published:** 2024-08-14

**Authors:** Feng Wang, Ying Wang, Shumei Liu, Linlin Cui, Feimeng Li, Xiaohe Wang

**Affiliations:** ^1^Department of Health Management and Policy, School of Public Health, Hangzhou Normal University, Hangzhou, Zhejiang, China; ^2^Hangzhou International Urbanology Research Center and Center for Urban Governance Studies, Hangzhou, Zhejiang, China; ^3^Department of Surgical Nursing, The First People’s Hospital of Yuhang District, Hangzhou, Zhejiang, China; ^4^Department of Surgical Nursing, Liangzhu Branch, The First Affiliated Hospital, Zhejiang University School of Medicine, Hangzhou, Zhejiang, China; ^5^Department of Preschool Education, Zhejiang Philosophy and Social Science Laboratory for Research in Early Development and Childcare, Jing Hengyi School of Education, Hangzhou Normal University, Hangzhou, Zhejiang, China

**Keywords:** parental migration, left-behind children, depression, non-suicidal self-injury, suicidal ideation

## Abstract

**Background:**

Parent–child separation raises concerns for the well-being of 69 million left-behind children (LBC) in China. However, the effects of parental migration status, timing of migration, and migration duration on the health of children remain unclear. This study aims to explore the association between different parent–child separation experience and a range of health outcomes in rural Chinese children.

**Method:**

A sample of 2,355 students, grades 5 to 8, from two provinces in China were recruited. Standardized self-report instruments collected data on demographics, separation status, and children’s health conditions.

**Results:**

Full data were available for 274 children with both parents currently migrating (BLBC), 638 children with one parent currently migrating (SLBC), 658 children with parents previously migrated (PLBC) and 785 children with non-migrating parents (NLBC). Regression model results showed that, compared to the NLBC group, BLBC and PLBC exhibited lower self-rated health (*p* < 0.05), higher depression (*p* < 0.05), and higher rates of non-suicidal self-injury behaviors (*p* < 0.05) and suicidal ideation (*p* < 0.05). Children who experienced parental separation before the age of three were at a higher risk for four health indicators. Additionally, children left behind by parents for more than 7 years had significantly worse health outcomes.

**Conclusion:**

Children who have experienced both current and previous parental migration, as well as earlier parental migration age and longer migration duration, are at a disadvantage in terms of health. These findings highlight the need for targeted interventions focusing on the most vulnerable children.

## 1 Introduction

Massive population movements, both internationally and internally, have significantly impacted core family structures and migrants’ hometowns over the past decades (1). According to the World Migration Report 2020, there were 272 million international migrants worldwide, accounting for about 3.5% of the global population (2). Additionally, 763 million people live within their own country but outside their hometowns (2). However, due to financial constraints, stringent immigration policies, and limited access to public services in destination cities, most migrating parents choose to leave their children in their hometowns (3). Consequently, hundreds of millions of children are left behind under the care of their extended families globally. Based on the available data, left-behind children are predominantly concentrated in low and middle-income countries, particularly in Asia, Eastern Europe, Latin America and Africa, with Asia accounting for the largest proportion of these children (4). China exemplifies this phenomenon, with large-scale internal migration resulting in 68.8 million left-behind children (LBC), representing 25% of the total number of Chinese children (5). The family systems theory offers a valuable perspective on the development of children in migrant families (6). According to this theory, parental migration can have both positive and negative impacts on children’s adjustment, as it involves a balance between increased family income and reduced parental care. While the additional income from remittances can enhance children’s nutrition and overall health, the absence of parents may result in decreased care, stimulation, and communication, potentially giving rise to psychological and behavioral problems. The literature has also paid significant attention to the effects of domestic and international parental migration on children’s health, focusing particularly on physical and mental health outcomes (7). However, the evidence about the health status of LBC remains conflicting. The systematic review of Fellmeth et al. (7), which included 111 studies, demonstrated that parental migration has adverse effects on the LBC’s health, with no evidence supporting any benefit. In comparison to non-LBC, LBC report significantly increased levels of anxiety, depression and suicidal thoughts. They are also more likely to suffer from wasting, stunting, and more substance use. Conversely, other reviews have found that LBC perform equally well or even better than non-LBC in various health, nutrition, and education indicators (8, 9). The impact of labor migration on the health of LBC has yet to research a consensus among scholars. One potential explanation for these mixed findings could be the influence of various mediating or moderating factors, such as the types of parents migrating, the age at which the child is left behind, and the duration of parental absence (7, 9).

In most circumstances, previous studies have regarded LBC as a single group. The differences among children with one migrating parent (SLBC), those with both parents migrating (BLBC), and those with parents who have previously migrated (PLBC) have received less attention. A small number of surveys have started to assess these differences, but the results are inconsistent. While some studies indicate that BLBC and SLBC may be more prone to depression compared to non-LBC (10, 11), others have discovered that the incidence of anxiety disorders for non-LBC is higher than that of BLBC or SLBC (12, 13). A possible explanation may be that these studies concentrated on different age ranges. Furthermore, these studies have been unable to provide information on how the mental health problems of LBC evolve when the family environment undergoes significant changes, such as reunions with their parents. There is a current lack of information regarding the effects of diverse parental migration patterns on the health of LBC.

There is lack of evidence to suggest the impact of age on one’s health upon separation from parents. According to the attachment theory, the initial bonds children form with their caregivers significantly influence their development (14). Typically, children first attach to their mothers or primary caregivers, with the possibility of forming additional attachments over time (15). Disruption of these attachments, referred to as the “strange situation,” can result in children experiencing feelings of depression, anxiety, or anger when they were unable to maintain proximity (15). Previous research indicates that the timing of parental migration can be highly significant to children. For instance, Liu et al. identified that parental migration before the age of 3 is a risk factor for anxiety and depression (16). Subsequent studies have demonstrated that being left-behind before 6 months old could lead to delays in early child development (17). However, a survey conducted in rural Central China revealed that the age of the children upon separation from their parents had no impact on their mental health (18). These inconsistent findings suggest that the above studies focus on relatively narrow age ranges and fail to capture the broader impact of parental migration timing on children’s health. For instance, some parents may opt to migrate for employment only after their children have started primary or junior high school, and the impact on their children’s health may differ from that of parents who migrate before their children reach 3 years of age. The duration of parental migration is a significant factor in determining the health of their children. According to the theory of cumulative (dis)advantage, individuals’ life experiences are shaped by the accumulation of early (dis)advantage (19, 20). Some researchers argue that long-term exposure to adverse environments may harm children’s health outcomes (21, 22). For example, Li et al. utilized the Chinese version of the Age and Stages Questionnaires and showed that prolonged maternal separation was linked to a higher likelihood of delays in social–emotional growth and fine motor skills (17). Drawing upon a unique nationally representative longitudinal dataset, Sun et al. found that longer maternal migration duration increases the likelihood of children being anemic (23). However, existing studies have primarily focused on the association between the duration of parental migration and the early childhood developmental outcomes, while research on the correlation between the length of separation from parents and the mental health of left-behind children remains comparatively scarce.

The objective of this research is to examine the various effects of parental migration status (BLBC, SLBC, PLBC), the timing of parental migration, and migration duration on the self-rated health, depression, and non-suicidal self-injury and suicidal ideation of children in rural China.

## 2 Methods

### 2.1 Participants and procedures

For ease of sampling, we aimed to select areas where there were large numbers of LBC. To accomplish this, we obtained child population figures from the 2020 National Census Survey. We then selected one county in southeast Anhui (Nanling) and one county in western Zhejiang province (Kaihua), with high proportions of LBC in rural areas. Anhui is a less wealthy inland province in central China, while Zhejiang is a developed coastal province. However, its western regions, such as Kaihua county, have a poorly developed economy. Three towns were randomly selected from each county, resulting in a total of six towns. In each town, one primary school and one middle school were chosen at random. Students from grades 5–6 (primary school) and grades 7–8 (middle school) in each school were invited to participate. To ensure that the respondents had the necessary reading comprehension to complete the survey, children from grades 1–4 were excluded. Furthermore, this study specifically focused on early adolescence (grades 5–8), as this period is commonly regarded as the starting point for dramatic physical and cognitive changes, as well as transitions in social status (24). A total of 3,025 students from 12 schools across Anhui and Zhejiang were selected to participate. Out of these, 2,931 students agreed to take part and completed the questionnaire, resulting in a response rate of 96.9%. However, another 90 students were deemed ineligible due to incomplete key variables, such as parental migration status and left behind characteristics. As other forms of parental absence may have varying effects on children, those whose parents divorced or died were excluded from the study (n = 486)We obtained permission from the local county education authorities to conduct the survey. Informed consent was obtained from both the eligible students and their parents or guardians before the survey. If the students agreed to participate, they were then asked to complete a self-filled survey in the classroom, without the presence of any teachers or school administrators. The students were informed that their participation would be kept confidential and anonymous, and they had the option to decline any questions and terminate the survey at any time.

### 2.2 Measures

#### 2.2.1 Socio-demographic variables

Socio-demographic characteristics evaluated in this study include gender (male/female), grade (grades 5–6/grades 7–8), parental education level (primary school or lower, middle school, high school or above), number of siblings, and province (Anhui and Zhejiang). Economic status was measured based on our previous research: “How do you feel your household wealth compares with the average in your community (13)?”

#### 2.2.2 Parental migration status and left-behind characteristics

To identify parental migration status, participants were asked: “Has your father (and/or mother) migrated to another city for work and been absent for over six months?” The responses were “no, never,” “yes, previously migrated,” and “yes, currently migrates.” If both parents were currently migrating, the participant was identified as a “BLBC”; if not, and if either parent was currently migrating, the participant was identified as an “SLBC”; if not, and if one or both parents previously migrated, the participant was identified as a “PLBC”; and if both parents had not migrated, the participant was identified as an “NLBC.” Ultimately, the study sample comprised 2,355 students, comprising 274 BLBC, 638 SLBC, 658 PLBC, and 785 NLBC.

Based on commonly accepted developmental stages in China, the age of a child when a parent first migrated was classified into four categories: (1) below 3 years, (2) 4 to 6 years, (3) 7 to 9 years, (4) 10 years or older (16). The duration of separation from parents was evaluated based on four level of responses: within 3 years, between 4 and 6 years, between 7 and 9 years, and exceeding 10 years (25).

#### 2.2.3 Depression

Child mental health was measured with the Chinese version of the Children’s Depression Inventory (CDI) (26, 27). The CDI is designed to detect depressive symptoms in children over the last 2 weeks and is one of the most widely used screening tools globally. The instrument consists of 27 self-administered items, each of which is scored between 0 and 2, with a total score ranges from 0 to 54. A high score signifies a greater degree of depression. A score of 19 or above was considered indicative of depression (26). The Cronbach’s α for the CDI in the current study was 0.894.

#### 2.2.4 Self-rated health

The self-rated health (SRH) was assessed through the question: “Overall, how is your health condition?” Responses were classified into “1 = extremely good,” “2 = very good,” “3 = good,” “4 = normal,” and “5 = not good” (28). In this study, responses with “extremely good” and “very good” were coded as 0, while “good,” “normal” and “not good” were coded as 1.

#### 2.2.5 Non-suicidal self-injury

Non-suicidal self-injury (NSSI) was measured using a question from the Composite International Diagnostic Interview, asking about the past 12 months: “Did you hurt yourself deliberately without suicide intent (any form of following behaviors: hitting, biting, cutting, burning, scratching, pinching, head banging and pulling hair) (29)?” There are two available answers to the question: “no” and “more than once.” Then “more than once” was recognized to be a cut-off of defining NSSI in the present study (24).

#### 2.2.6 Suicidal ideation

The Beck Depression Inventory (BDI) was used to assess suicidal ideation (SI) with one item, which is a self-report questionnaire widely used for the screening of depression (30). Previous research suggests that utilizing a single item from the BDI may be an effective for measuring SI (31). Depended on participants’ answers to the SI, they were divided into four groups: (1) “I do not have any thoughts of killing myself,” (2) “I have thoughts of killing myself but I would not carry them out,” (3) “I would like to kill myself” and (4) “I would kill myself if I had the chance.” The four options were available to respondents, and those who selected (2), (3), and (4) were subsequently classified as having SI.

### 2.3 Statistical analysis

Firstly, we employed the chi-square tests to compare socio-demographic characteristics of four groups of children with diverse parental migration statuses (BLBC, SLBC, PLBC, and NLBC). Secondly, bivariate associations of dependent variables (SRH, CDI, NSSI, and SI) with different characteristics of parental separation were evaluated using chi-square tests. Finally, for those dependent indicators that exhibited statistical significance in the univariate analysis, we controlled for gender, grade, household wealth level, parental education level, only child status, and province using binary logistic regression models. To prevent collinearity, we used logistic regression models separately to analyze parental migration status, age at separation and duration of separation. A *p*-value of <0.05 was regarded as statistically significant.

## 3 Results

Table 1 displays the descriptive statistics of children based on their parents’ migration status. In total, there were slightly fewer girls (49.2%) than boys (50.8%) among the respondents, but this difference was not observed among the four groups. The number of students in grades 7–8 was similar among the groups. Over 30% of NLBC came from wealthier households, whereas only 22.3% of SLBC did. When it comes to the highest level of parental education, parents of NLBC (35.6%) were more likely than SLBC (25.9%) and PLBC (28.9%) to have a high school degree or higher. In the full sample, approximately one-third of participants were the only child in their family. However, the percentage of only children was highest among the BLBC group (33.9%) and lowest among the NLBC group (24.4%). In our sample, 50.1% of children were recruited in Anhui and 49.9% in Zhejiang, with a higher proportion of children in Zhejiang (57.3%) being BLBC.

**Table 1:** Socio-demographic of study participants by parental migration status, *n*(%).

	BLBC	SLBC	PLBC	NLBC	χ^2^	*p*-value
	*n* = 274	*n* = 638	*n* = 658	*n* = 785		
Gender					5.96	0.114
Male	131(48.9)	342(54.9)	321(50.2)	375(48.8)		
Female	137(51.1)	281(45.1)	319(49.8)	394(51.2)		
Grade					3.85	0.279
Grade 5–6	135(49.3)	307(48.1)	295(44.8)	391(49.8)		
Grade 7–8	139(50.7)	331(51.9)	363(55.2)	394(50.2)		
Household wealth level					15.85	0.015
Much better off/better off	73(26.7)	142(22.3)	161(24.5)	240(30.7)		
The same	180(65.9)	436(68.6)	446(67.9)	493(63.0)		
Poorer/much poorer	20(7.3)	58(9.1)	50(7.6)	50(6.4)		
Parental education level					19.06	0.004
Primary school or lower	21(7.7)	71(11.2)	75(11.4)	77(9.8)		
Middle school	163(60.1)	400(62.9)	392(59.7)	428(54.6)		
High school or above	87(32.1)	165(25.9)	190(28.9)	279(35.6)		
Only child					10.19	0.017
Yes	93(33.9)	184(28.9)	185(28.2)	190(24.4)		
No	181(66.1)	453(71.1)	470(71.8)	590(75.6)		
Province					9.51	0.023
Anhui	117(42.7)	329(51.6)	350(53.2)	384(48.9)		
Zhejiang	157(57.3)	309(48.4)	308(46.8)	401(51.1)		

LBC, left-behind children; BLBC, left-behind children with both parents migrating; SLBC, left-behind children with one parent migrating; PLBC, left-behind children with one or both parents previously migrated; NLBC, neither parent had migrated.

Table 2 illustrates the percentages of self-rated health, depression, NSSI and SI across different groups of parental migration and left-behind characteristics. There were significant between-group differences in all four kinds of health outcomes. BLBC reported the highest rates of worse self-rated health (40.9%, *p* < 0.001), depression (28.1%, *p* < 0.001), NSSI (24.3%, *p* = 0.013), and SI (34.8%, *p* < 0.001). As also shown in Table 2, children who left behind by parents before their third birthday had the lowest self-rated health (36.4%, *p* = 0.001), highest depression (29.4%, *p* < 0.001), NSSI (27.0%, *p* = 0.002), and SI (35.2%, *p* < 0.001). With regard to the duration of separation from parents, the proportion of lower self-rated health was higher among those who were left behind for 3 years (30.5%) than that among those who never had left-behind experience (26.2%) and further increased among those who were left behind for 4–6 years (33.5%), 7–9 years (38.3%), and more than 10 years (36.0%). The trends for higher depression, NSSI, and SI with the length of parental migration were similar.

**Table 2:** Association between left-behind characteristics and health outcomes.

	Self-rated health^#^		Depression^$^		NSSI^%^		SI^&^	
	*n*(%)	*p*-value	*n*(%)	*p*-value	*n*(%)	*p*-value	*n*(%)	*p*-value
Parental migration		<0.001		<0.001		0.013		<0.001
BLBC	112(40.9)		77(28.1)		66(24.3)		95(34.8)	
SLBC	211(33.1)		147(23.0)		134(21.1)		173(27.2)	
PLBC	217(33.0)		147(22.3)		159(24.3)		214(32.7)	
NLBC	205(26.1)		130(16.6)		139(17.8)		185(23.6)	
Age of the child when a parent first migrated		0.001		<0.001		0.002		<0.001
Never	205(26.2)		130(16.6)		139(17.8)		185(23.6)	
≤3	208(36.4)		168(29.4)		153(27.0)		200(35.2)	
4–6	159(33.4)		90(18.9)		102(21.5)		142(29.8)	
7–9	102(33.1)		66(21.4)		63(20.5)		86(27.9)	
≥10	68(33.2)		44(21.5)		40(19.5)		51(24.9)	
Length of parental migration		<0.001		<0.001		0.005		0.001
Never	205(26.2)		130(16.6)		139(17.8)		185(23.6)	
≤3	130(30.5)		90(21.1)		90(21.2)		118(27.8)	
4–6	138(33.5)		77(18.6)		84(20.4)		122(29.5)	
7–9	133(38.3)		80(23.1)		82(23.8)		111(32.2)	
≥10	134(36.0)		122(32.8)		101(27.2)		130(34.9)	

^#^0 = extremely good/very good, 1 = good/normal/not good; $: 0 = no, 1 = yes (total score ≥ 19);%NSSI (non-suicidal self-injury): 0 = 0 time, 1 = ≥1 times; &SI (suicidal ideation): 0 = I do not have any thoughts of killing myself, 1 = I have thoughts of killing myself but I would not carry them out/I would like to kill myself/I would kill myself if I had the chance.

Table 3 summarizes the results of logistic regression analysis for parental migration status and left behind characteristics on children’s health. After adjusting for socio-demographic variables, BLBC and PLBC had worse self-rated health (AOR = 1.89, 95%CI = 1.39–2.57, *p* < 0.001; 1.33, 1.04–1.69, *p* < 0.05), higher depression (2.10, 1.50–2.94, *p* < 0.001; 1.43, 1.09–1.89, *p* < 0.05), higher NSSI (1.51, 1.07–2.12, *p* < 0.05; 1.47, 1.13–1.91, *p* < 0.01) and SI (1.73, 1.27–2.37, *p* < 0.01; 1.55, 1.22–1.98, *p* < 0.001). Furthermore, SLBC had worse self-rated health and higher depression than NLBC, but not higher NSSI and SI. When compared with children who never had left-behind experience, those who experienced parental separation before 3 years old were more likely to have worse self-rated health (1.56, 1.21–1.99, *p* < 0.001), higher depression (2.09, 1.59–2.74, *p* < 0.001), higher NSSI (1.70, 1.30–2.23, *p* < 0.001) and SI (1.79, 1.39–2.30, *p* < 0.001). In regard to the length of parental migration, children who reported 7 years or longer duration of parental separation (7–9 years and ≥ 10 years) were more likely to report worse self-rated health (1.72, 1.29–2.28, *p* < 0.001; 1.44, 1.08–1.90, *p* < 0.05), higher depression (1.55, 1.12–2.14, *p* < 0.01; 2.44, 1.81–3.29, *p* < 0.001), higher NSSI (1.48, 1.08–2.04, *p* < 0.05; 1.68, 1.24–2.27, *p* < 0.01) and SI (1.56, 1.16–2.09, *p* < 0.01; 1.72, 1.29–2.28, *p* < 0.001).

**Table 3:** Health outcomes and left-behind characteristic associations, AOR (95%CI).

	Self-rated health	Depression	NSSI	SI
**Parental migration (ref: NLBC)**^**#**^				
BLBC	1.89(1.39,2.57)^***^	2.10(1.50,2.94)^***^	1.51(1.07,2.12)^*^	1.73(1.27,2.37)^**^
SLBC	1.37(1.07,1.74)^*^	1.55(1.18,2.04)^**^	1.24(0.94,1.63)	1.23(0.96,1.58)
PLBC	1.33(1.04,1.69)^*^	1.43(1.09,1.89)^*^	1.47(1.13,1.91)^**^	1.55(1.22,1.98)^***^
**Age of the child when parents migrated (ref: never)**^**$**^**, year**				
≤3	1.56(1.21,1.99)^***^	2.09(1.59,2.74)^***^	1.70(1.30,2.23)^***^	1.79(1.39,2.30)^***^
4–6	1.33(1.03,1.74)^*^	1.21(0.89,1.64)	1.26(0.94,1.70)	1.36(1.04,1.78)^*^
7–9	1.40(1.04,1.90)^*^	1.48(1.06,2.09)^*^	1.25(0.89,1.76)	1.28(0.94,1.75)
≥10	1.30(0.92,1.84)	1.33(0.90,1.97)	1.04(0.69,1.56)	1.01(0.70,1.47)
**Length of parental migration (ref: never)**^**&**^**, years**				
≤3	1.16(0.88,1.52)	1.34(0.98,1.82)	1.22(0.90,1.65)	1.18(0.89,1.57)
4–6	1.48(1.12,1.95)^**^	1.24(0.90,1.71)	1.22(0.89,1.67)	1.44(1.09,1.91)^*^
7–9	1.72(1.29,2.28)^***^	1.55(1.12,2.14)^**^	1.48(1.08,2.04)^*^	1.56(1.16,2.09)^**^
≥10	1.44(1.08,1.90)^*^	2.44(1.81,3.29)^***^	1.68(1.24,2.27)^**^	1.72(1.29,2.28)^***^

Adjusted for gender, grade, household wealth level, parental education level, only child and province. ^*^p < 0.05, ^**^p < 0.01, ^***^p < 0.001. NSSI, non-suicidal self-injury; SI, suicidal ideation. ^#^Self-rated health: Cox & Snell R^2^ = 0.078; Nagelkerke R^2^ = 0.109; Depression: Cox & Snell R^2^ = 0.041; Nagelkerke R^2^ = 0.064; NSSI: Cox & Snell R^2^ = 0.032; Nagelkerke R^2^ = 0.049; SI: Cox & Snell R^2^ = 0.061; Nagelkerke R^2^ = 0.087. ^$^Self-rated health: Cox & Snell R^2^ = 0.077; Nagelkerke R^2^ = 0.108; Depression: Cox & Snell R^2^ = 0.046; Nagelkerke R^2^ = 0.072; NSSI: Cox & Snell R^2^ = 0.034; Nagelkerke R^2^ = 0.052; SI: Cox & Snell R^2^ = 0.063; Nagelkerke R^2^ = 0.091. ^&^Self-rated health: Cox & Snell R^2^ = 0.078; Nagelkerke R^2^ = 0.109; Depression: Cox & Snell R^2^ = 0.047; Nagelkerke R^2^ = 0.073; NSSI: Cox & Snell R^2^ = 0.033; Nagelkerke R^2^ = 0.051; SI: Cox & Snell R^2^ = 0.060; Nagelkerke R^2^ = 0.087.

## 4 Discussion

In the modern era, China’s rapid economic growth and social transformation have resulted in a significant number of children whose parents migrate to larger cities in pursuit of better employment and living opportunities. There is an urgent need to comprehend the health issues of these children. This paper aims to explore the varying effects of parental migration patterns, the timing of parental migration, and migration duration on the health of LBC by utilizing data from two Chinese provinces with differing levels of socio-economic development. We have demonstrated that left-behind children, including BLBC, SLBC, and PLBC, are significantly more likely to report lower levels of self-rated health and higher levels of depression than NLBC. After adjusting for all covariates, it appeared that BLBC and PLBC were more disadvantaged in terms of NSSI and SI compared to NLBC. Additionally, we noted a notable impact of the child’s age at separation from parents and the length of parental migration on the four health outcomes indicators.

Firstly, we confirm the findings of prior research that children whose parents have both migrated had the lowest self-rated health, highest levels of depression, NSSI, and SI among the four groups of rural children (32). However, children of parents who have migrated alone reported NSSI and SI prevalence rates similar to those of NLBC (33). There are several possible explanations for this result. The well-being of children is primarily affected by the trade-off between an increase in family income and a reduction in parental care. On one hand, increasing income through remittances may positively impact nutrition and general health. On the other hand, the lack of parental oversight could result in reduced care and supervision, resulting in cognitive delays and the emergency of health issues. Children of parents who migrate alone may face improved financial circumstances due to the earnings of their migrating parent. And they can also benefit from staying with one of their residing parents. However, due to the reduction of parental supervision and the weakening of parent–child attachment and communication, both father and mother’s migration may be more harmful (25, 32). Importantly, our research showed that after adjusting for major confounding factors, the negative impact of previous separation from migrant parents on children’s health is similar to that of the current absence of both parents. This may be due to a number of potential explanations. Through our interviews with 17 migrant parents in rural Zhejiang Province, we discovered that some parents have decided to return permanently due to the advancement of their children or the occurrence of some concerning events (34). The return of parents may even bring new challenges to children’s lives due to the change of their primary caregivers (35). Future research needs to elaborate on the specific risks faced by PLBC.

Secondly, an important finding of this work is that early parental migration is linked with a heightened risk of poorer self-rated health, greater levels of depression, and higher rates of NSSI and SI. Children left behind by parents before their third birthday had the worst self-rated health and mental health. Our findings align with prior research that underscores the importance of parent–child attachment and the detrimental effects of early parental migration (16). The theory of attachment may aid in explaining the impact of migration age on the health of children. The initial 2 years of life serve as a critical period for the development of attachment (14). During this crucial period, children experienced repeated separations and short reunions with their parents, disrupting the formation of attachment (36). The theory suggests that children with secure attachments develop better emotional functioning, while disruptions in these attachments lead to increased insecurity and ambiguous loss (37). This disruption affects psychosocial well-being throughout childhood and adolescence (38). Studies on early childhood development underlines the critical importance of the “first 1,000 days.” Experts highlight that reduced dietary diversity and lack of stimulating activities during this period can negatively impact future outcomes, including educational attainment, income, and health (39, 40).

Thirdly, our research indicated that parent–child separation, especially long-term separation, can negatively affect the health of LBC. The study showed that LBC individuals who had been left behind by their parents for 4–6 years were more likely to experience poorer self-rated health and a higher likelihood of developing SI compared to those who had never experienced left-behind syndrome. With increased migration duration, the influence of parental migration grows. The risks of worse self-rated health, depression, NSSI, and SI progressively increased with longer parental migration duration (≥7 years). Previous studies have also found the cumulative formation of health outcomes and the significant impact of long-term exposure to parental migration (22). It is possible that rural LBC in China receive relatively less parental attention, care, and supervision when their parents are absent. Parental investments, including attention, care, and supervision have been recognized as key resources for adequate development and maturity (41). However, the impact of parental migration duration on children’s health is still a limited topic of research, warranting further attention.

The present study has several limitations. First, the timing of parental migration and migration duration was collected through students’ self-report questionnaires, which could be affected by recall bias. However, parents/guardians received a letter 2 days before the survey. They were invited to provide some key information including parents’ migration status, the timing of parental migration and migration duration. Second, we were unable to investigate disparities in health outcomes between father-only migration and mother-only migration due to the limited number of mother-only cases in our sample (2.1%). Third, our data on family income was derived from the subjective perceptions of children’s economic conditions, rather than objective measurements. Although children may be unaware of their parents’ annual income, the financial status of migrant parents could potentially serve as a confounding factor, as poorer parents may leave their children at a younger age and for a longer duration to seek urban employment (16). Fourth, the use of a cross-sectional design in researching results from only two provinces in rural China must be approached with caution. In future research, it is expected that a longitudinal design will be employed to elucidate the complex impacts of parental migration on children’s health. Fifth, this study only evaluated a limited range of potential determinants; other variables like the quality and forms of caregiving were not included.

Despite the limitations, this research has made a remarkable contribution to our understanding of the impact of migration on children’s health by comparing BLBC, SLBC, PLBC and NLBC, and by exploring the impacts of parental migration time and duration. According to this study, children who have both parents currently migrating and who were previously left behind are the most vulnerable children. Our results also suggest that children left behind at a younger age (≤ 3 years old) and for a longer duration (≥7 years) experience poorer self-rated health, more symptoms of depression, and higher levels of NSSI and SI. The China National Program of Action for Child Development (2021–2030) underscores the necessity of strengthening mental health services for children, particularly emphasizing the attention to and fulfillment of the psychological development needs of left-behind children, while simultaneously improving the abilities of teachers and caregivers in preventing and identifying psychological and behavioral abnormalities among children (42). Given the vast number of left-behind children and taking into account our research findings, it is imperative that government, schools, communities, and caregivers prioritize their attention on these most vulnerable subgroups of left-behind children.

## Data availability statement

The data that support the findings of this study are available from the corresponding author upon reasonable request.

## Ethics statement

The studies involving humans were approved by the Ethics Committees of the School of Public Health of Zhejiang University (Number: ZGL202006-11). The studies were conducted in accordance with the local legislation and institutional requirements. Written informed consent for participation in this study was provided by the participants’ legal guardians/next of kin.

## Author contributions

FW: Conceptualization, Data curation, Formal analysis, Funding acquisition, Writing – original draft. YW: Data curation, Project administration, Writing – review & editing. SL: Data curation, Project administration, Writing – review & editing. LC: Methodology, Writing – review & editing. FL: Data curation, Project administration, Writing – review & editing. XW: Supervision, Writing – review & editing.
